# Supplementary figures and images for: Early Pregnancy Modulates Expression of the Nod-like Receptor Family in Lymph Nodes of Ewes
Source: Animals (Basel). 2022 Nov 25;12(23):3285. doi: 10.3390/ani12233285 (PMC9738492; doi:10.3390/ani12233285)

Figure S1 Original Western Blot Figure for Figure 2

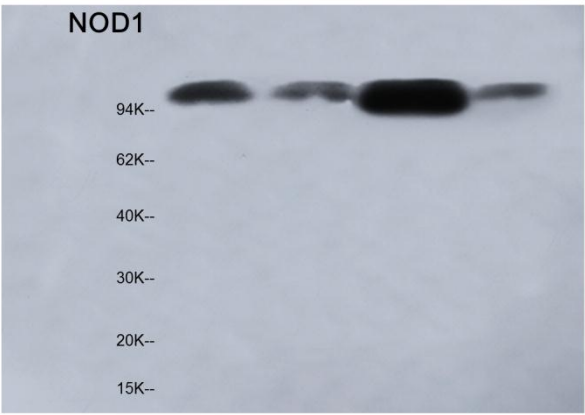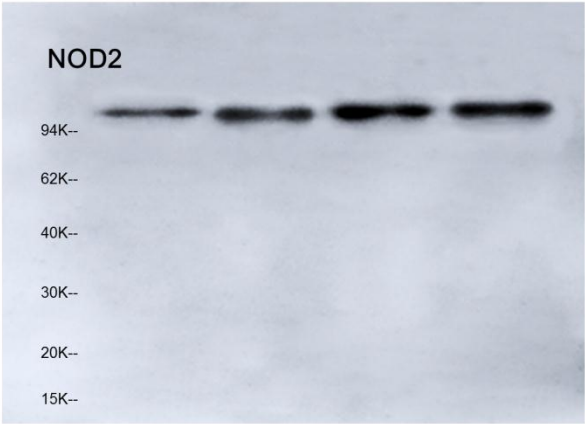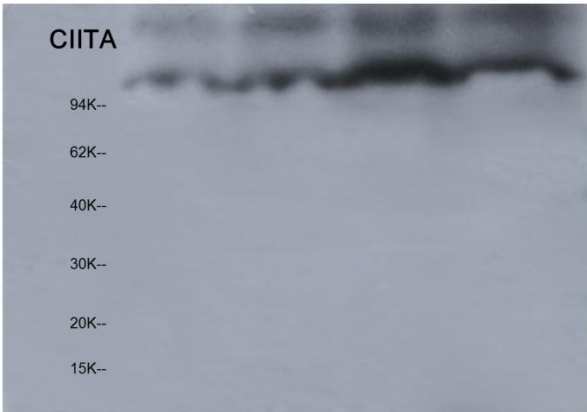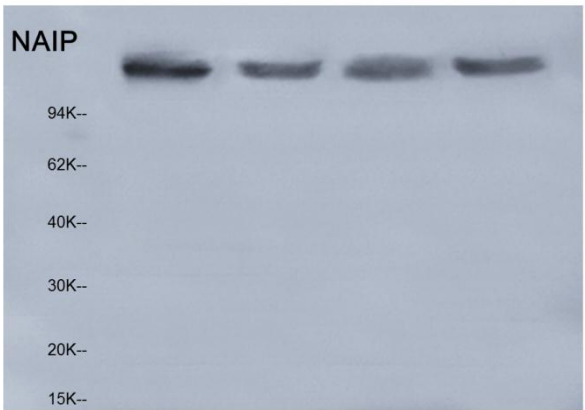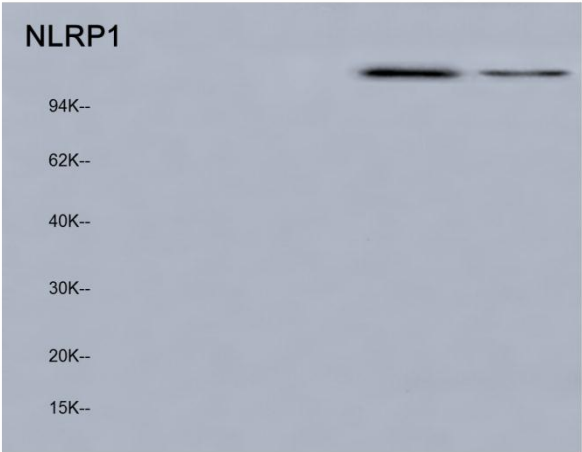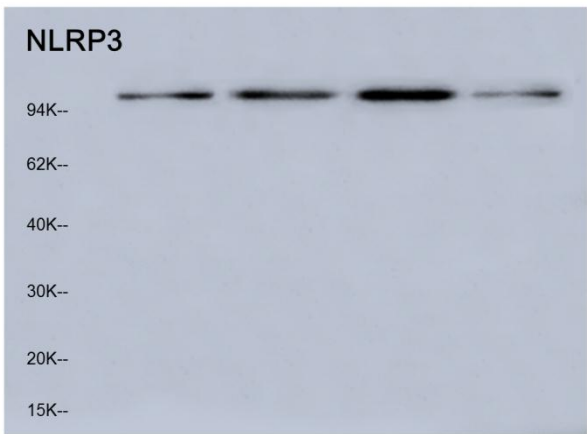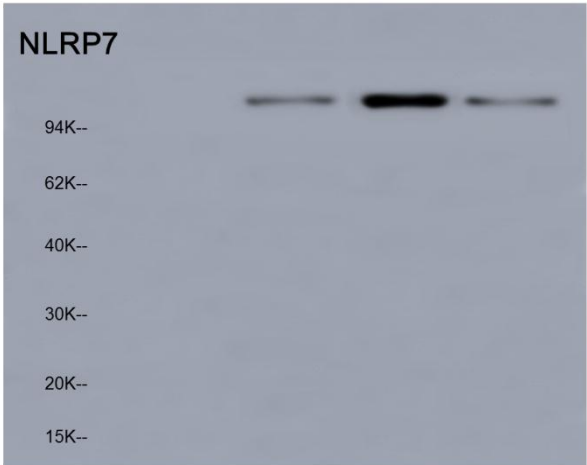

Supplement: Supplementary file 1 [file animals-12-03285-s001.zip › animals-1987735-Supplementary Materials.pdf]
